# The impact of the 2009 influenza pandemic on the seasonality of human respiratory syncytial virus: A systematic analysis

**DOI:** 10.1111/irv.12884

**Published:** 2021-07-04

**Authors:** You Li, Xin Wang, Takondwa Msosa, Femke de Wit, Jayne Murdock, Harish Nair

**Affiliations:** ^1^ Centre for Global Health, Usher Institute University of Edinburgh Edinburgh UK; ^2^ School of Public Health Nanjing Medical University Nanjing China

**Keywords:** COVID‐19, influenza virus, pandemic, respiratory syncytial virus, seasonality

## Abstract

**Background:**

Several local studies showed that the 2009 influenza pandemic delayed the RSV season. However, no global‐level analyses are available on the possible impact of the 2009 influenza pandemic on the RSV season.

**Objectives:**

We aim to understand the impact of the 2009 influenza pandemic on the RSV season.

**Methods:**

We compiled data from published literature (through a systematic review), online reports/datasets and previously published data on global RSV seasonality and conducted a global‐level systematic analysis on the impact of the 2009 influenza pandemic on RSV seasonality.

**Results:**

We included 354 seasons of 45 unique sites, from 26 countries. Globally, the influenza pandemic delayed the onset of the first RSV season by 0.58 months on average (95% CI: 0.42, 0.73; maximum delay: 2.5 months) and the onset of the second RSV season by a lesser extent (0.25 months; 95% CI: 0.12, 0.39; maximum delay: 3.4 months); no delayed onset was observed for the third RSV season. The delayed onset was most pronounced in the northern temperate, followed by the southern temperate, and was least pronounced in the tropics.

**Conclusions:**

The 2009 influenza pandemic delayed the RSV onset on average by 0.58 months and up to 2.5 months. This suggests evidence of viral interference as well as the impact of public health measures and has important implications for preparedness for RSV season during the ongoing COVID‐19 pandemic and future pandemics.

## 1 INTRODUCTION

Respiratory syncytial virus (RSV) is the most common pathogen identified in young children with acute lower respiratory infections^1^, ^2^ and poses a major burden on hospital beds during the peak of RSV transmission. RSV activity has clear seasonality in most parts of the world; RSV season, usually defined as a particular period of time with high RSV activity above a certain threshold, typically starts in late autumn and early winter in temperate regions and lasts for about 5 months.^3^ RSV seasonality information is important for health services planning as well as the timing of RSV passive prophylaxis.

The pandemic influenza H1N1 2009 virus was first detected in the United States in April 2009. It quickly spread globally since then, and the World Health Organisation (WHO) declared a pandemic in June 2009.^4^ In August 2010, WHO declared the end of the influenza pandemic.^5^

Several local reports from China,^6^, ^7^, ^8^ France,^9^ Germany^10^ and Israel^11^, ^12^ showed that the 2009 influenza pandemic delayed the RSV season by several weeks, whereas a study from Spain did not observe any differences in RSV season between the pandemic and the pre‐pandemic period (a summary of these reports is available in Table S1). However, no global‐level analyses are available. In the present study, we complied data from published literatures, online reports/datasets and previously published data on global RSV seasonality^3^ and conducted a global‐level systematic analysis on the impact of the 2009 influenza pandemic on RSV seasonality.

## 2 METHODS

### 2.1 Definitions

Based on whether an RSV season occurred during the 2009 influenza pandemic, we categorised RSV seasons into four different periods: pre‐pandemic, pandemic (1st RSV season), pandemic (2nd RSV season) and post‐pandemic. An RSV season was grouped into pre‐pandemic period if the entire season occurred before April 2009. An RSV season was grouped into pandemic (1st RSV season), if it was the first RSV season since April 2009 and was grouped into pandemic (2nd RSV season), if it was the second RSV season since April 2009. Lastly, an RSV season was grouped into post‐pandemic period if it was the 3rd or later RSV season since April 2009.

### 2.2 Data source

We collected RSV seasonality results (e.g. onset, offset, peak and duration of RSV season) and RSV seasonality data (e.g. weekly or monthly counts of laboratory‐confirmed RSV infection) from the literature via a systematic literature review, online datasets/reports and previously published data on global RSV seasonality.^3^ The following eligibility criteria were applied for the selection process.

#### 2.2.1 Inclusion criteria

- Studies reporting laboratory‐confirmed incidence data of human infection of RSV.
- RSV seasonality results or RSV seasonality data should be extractable for the pandemic (1st RSV season) period as defined above, plus at least 1 year in pre‐ or post‐pandemic period.
- Studies should be able to test RSV year‐round (e.g. not just during influenza seasons) and should report at least 25 positive RSV cases per year.^3^
- For studies that reported RSV seasonality data, the data should be made available at least on a monthly basis.

#### 2.2.2 Exclusion criteria

- Studies reporting respiratory infections only among those with special medical conditions (e.g. patients with chronic obstructive pulmonary disease or patients infected with human immunodeficiency virus).
- Studies only reporting nosocomial infections.

#### 2.2.3 Systematic literature review

A systematic literature review (PROSPERO registry number: CRD42021239011) was conducted. We searched three databases, Medline (Ovid), Embase (Ovid) and Global Health (Ovid) for any publications between 2009 and 2020 that potentially fulfilled the selection criteria above. The detailed search strategy can be found in Text S1. Publications in any languages were considered for eligibility. The literature search and screening (including title and abstract screening and full‐text screening) were conducted by two reviewers Y. L. and T. M., independently, with inconsistencies resolved through discussion among the review team. For data extraction, we used a standard data extraction form, modified based on our previous global seasonality of respiratory viruses study.^3^ The data extraction form collected information on study sites, period, subjects, case definition, clinical specimens, RSV testing method, RSV seasonality results (including onset, offset, peak and duration as per reported in the literature) and RSV seasonality data (e.g. weekly or monthly counts of RSV positives). The data extraction was conducted independently by T. M. and jointly by X. W., F. d. W. and J. M. Where any inconsistencies occurred, a final decision was made by Y. L.

#### 2.2.4 Additional data

We extracted RSV activity data from three online datasets/reports from the FluWatch programme in Canada,^13^ the Infectious Agents Surveillance Report in Japan^14^ and the Virology Annual Report in New Zealand^15^ between 2000 and 2019. No RSV data were extracted for 2020 considering the impact of COVID‐19 on RSV seasonality. We also included RSV activity data from our previously published review on global RSV seasonality.^3^

### 2.3 Quality assessment

For each included record, two reviewers (X. W. and T. M.) conducted quality assessment independently using a modified questionnaire based on our previous study.^3^ Briefly, the questionnaire comprised three brief questions regarding data representativeness, diagnostic practices and timely reporting; for each question, each study was rated from A (very good) to D (bad). Studies with any ‘D’ ratings were excluded from the analysis and studies with any ‘C’ ratings were excluded from the sensitivity analysis that is described in the next section. Details of the questionnaire are available in Text S2.

### 2.4 Data analysis

For those studies/reports/datasets that had RSV seasonality data (e.g. weekly/monthly counts of RSV positives), we determined the RSV seasonality results using the following approach: we first divided the timeline into 12‐month intervals so that each interval had a complete RSV season; for each of the weeks/months per interval, we then calculated the annual cumulative proportion (ACP), which ranged 0–1. The ACP of the last week/month of the interval should be 1. Based on ACP, RSV onset was defined as the week/month with ACP being 0.1, and RSV offset was defined as the week/month with ACP being 0.9. Linear interpolation was applied to allow for non‐integer results for RSV onset/offset (e.g. RSV onset could be month 1.2 or week 5.5 rather than month 1 or week 6). RSV duration was defined as the difference between RSV onset and offset. RSV peak was defined as the week/month with the highest RSV counts in each 12‐month interval.

For those studies that reported RSV seasonality results (e.g. onset, offset, peak and duration of RSV season), we used the extracted results for our data analysis. If studies had both RSV seasonality results and RSV seasonality data, we prioritised the inclusion of RSV seasonality data in our main analysis and prioritised the inclusion of RSV seasonality results in our sensitivity analysis. Where available, we calculated the time interval between onset and peak as an additional measure of interest.

Our primary outcome of interest, determined a priori, was the difference in RSV onset between the first RSV season in the pandemic and RSV seasons during the inter‐pandemic period (i.e. pre‐pandemic and post‐pandemic). Secondary outcomes of interest included the difference in RSV offset, peak, duration and onset‐to‐peak interval between the first RSV season in the pandemic and the inter‐pandemic period. The same comparisons as described above were repeated between the second RSV season since the pandemic and the inter‐pandemic period. An ad hoc analysis was also conducted to compare the difference in RSV onset between the third RSV season since the pandemic and the inter‐pandemic period. Subgroup analysis that separated pre‐ and post‐pandemic periods was also conducted. Based on the quality assessment results, we conducted a sensitivity analysis that excluded studies with ‘C’ ratings in any of the questions. We also conducted a sensitivity analysis that excluded studies reporting less than five RSV seasons. As an exploratory analysis, we compared the time length required to reach different levels of ACP between the pandemic and inter‐pandemic periods, by using seasonality data. This would help examine the impact of the influenza pandemic on RSV activity over the complete course of one RSV season.

Moreover, as latitude played an important role in RSV seasonality,^3^ we conducted stratified analysis by three latitude groupings: northern temperate (>23.44 degrees), tropics (between −23.44 and 23.44 degrees) and southern temperate (<−23.44 degrees).

All data analyses and visualisations were conducted using the R software (version 3.6.2).

## 3 RESULTS

**FIGURE 1:**
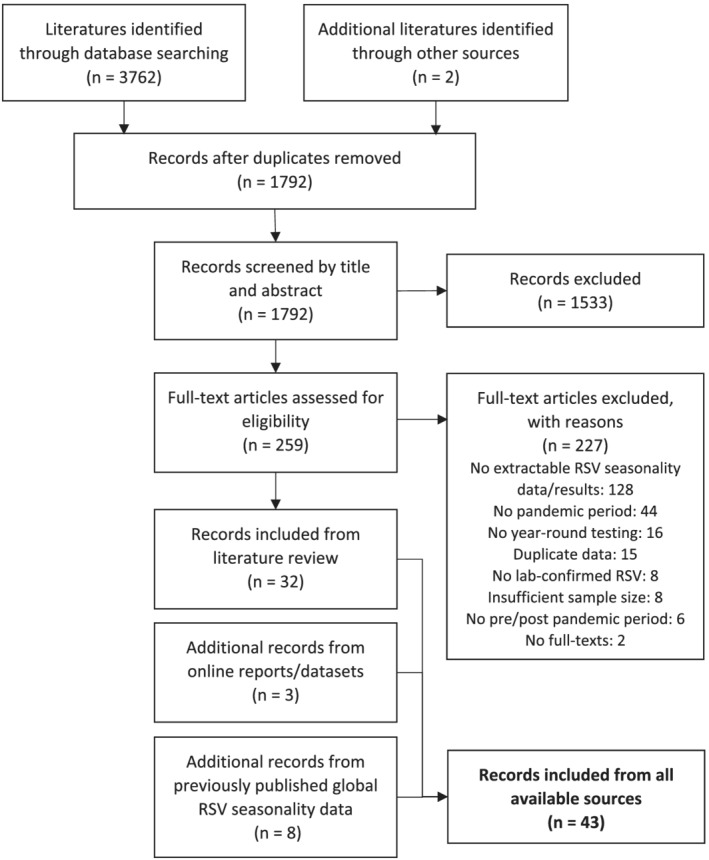
Flowchart presenting study selection process

As shown in Figure 1, we screened 1792 records by title and abstract and 259 records by full‐text, which led to the inclusion of 32 studies from the literature review. In addition, we included three more records from online datasets/reports and eight more records from previously published data on global RSV seasonality,^3^ bringing the total number of included records to 43. These 43 records provided data on 354 seasons for 45 unique sites (48 sites in total), from 26 countries. More detailed information on the included records is presented in Table S2.

Overall, we found that the influenza pandemic delayed the onset of the first RSV season by 0.58 months (95% CI: 0.42, 0.73) on average, with a maximum delay of 2.5 months. By comparison, the influenza pandemic delayed the onset of the second RSV season by a lesser extent, which was 0.25 months (95% CI: 0.12, 0.39), with a maximum delay of 3.4 months. RSV seasons during the pandemic were found to be shorter, and the interval between onset and peak was also shorter (Table 1). Similar findings were observed in the sensitivity analyses that prioritised the inclusion of seasonality results (Table S3); excluded studies with any ‘C’ ratings (Table S4); and excluded studies with less than five RSV seasons (Table S5). The above findings did not change substantively when only including the pre‐pandemic period or when only including the post‐pandemic period. In the ad hoc analysis that assessed the third RSV season (since the influenza pandemic), we did not observe a statistically significant delay in the RSV season onset (95% CI: −0.33, 0.04).

**TABLE 1:** Comparison of RSV seasonality between 2009 influenza pandemic and inter‐pandemic periods

Period	Onset		Peak		Onset‐peak interval		Offset		Duration	
	*N* of seasons	Difference in months	*N* of seasons	Difference in months	*N* of seasons	Difference in months	*N* of seasons	Difference in months	*N* of seasons	Difference in months
Pandemic (1st season) versus pre/post‐pandemic^a^	**238**	**0.58 (0.42, 0.73)**	**245**	**0.31 (0.12, 0.50)**	**213**	**−0.29 (−0.51, −0.07)**	**234**	**0.03 (−0.15, 0.19)**	**234**	**−0.55 (−0.77, −0.33)**
Pandemic (1st season) versus pre‐pandemic^a^	128	0.63 (0.40, 0.86)	127	0.25 (−0.05, 0.55)	108	−0.44 (−0.80, −0.08)	125	−0.07 (−0.31, 0.17)	125	−0.70 (−1.06, −0.35)
Pandemic (1st season) versus post‐pandemic^a^	110	0.51 (0.31, 0.72)	118	0.38 (0.16, 0.61)	105	−0.13 (−0.37, 0.10)	109	0.14 (−0.10, 0.37)	109	−0.37 (−0.62, −0.13)
Pandemic (2nd season) versus pre/post‐pandemic^a^	**202**	**0.25 (0.12, 0.39)**	**209**	**0.26 (0.05, 0.46)**	**177**	**−0.25 (−0.45, −0.05)**	**198**	**−0.13 (−0.31, 0.06)**	**198**	**−0.40 (−0.57, −0.22)**
Pandemic (2nd season) versus pre‐pandemic^a^	97	−0.02 (−0.18, 0.15)	96	0.20 (−0.13, 0.53)	77	−0.15 (−0.52, 0.22)	94	−0.29 (−0.60, 0.02)	94	−0.29 (−0.58, 0.002)
Pandemic (2nd season) versus post‐pandemic^a^	105	0.50 (0.29, 0.71)	113	0.30 (0.06, 0.54)	100	−0.32 (−0.54, −0.11)	104	0.021 (−0.19, 0.23)	104	−0.49 (−0.70, −0.28)

*Note*: In bold are the main results.

^a^ Pandemic (1st season) is defined as the first RSV season since April 2009. Pandemic (2nd season) is defined as the second RSV season since April 2009.

**FIGURE 2:**
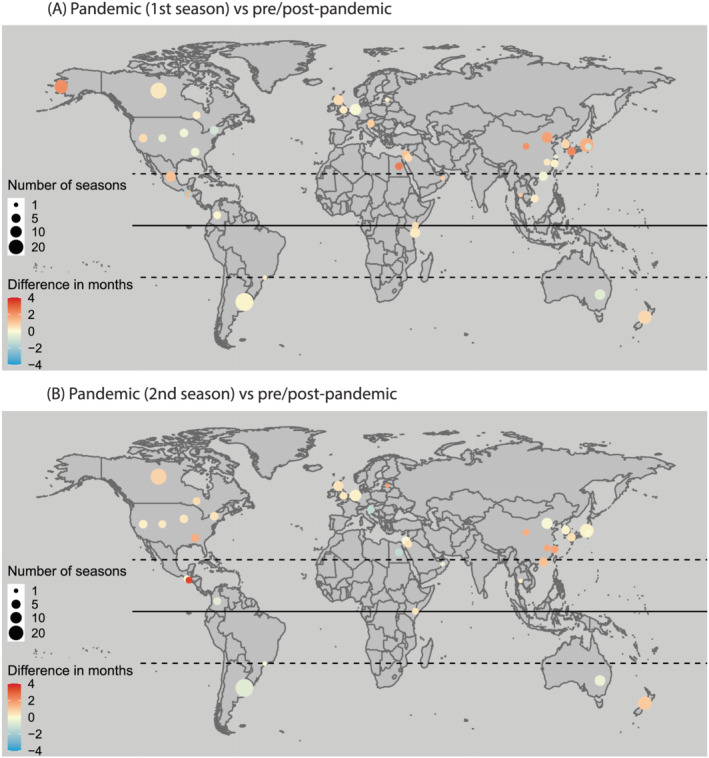
Comparison of RSV onset between 2009 influenza pandemic and inter‐pandemic periods by study site. Reference is pre/post‐pandemic period. Pandemic (1st season) is defined as the first RSV season since April 2009. Pandemic (2nd season) is defined as the second RSV season since April 2009

Some regional variations in the effects of the influenza pandemic on RSV seasonality were noted, as shown in Figure 2 and Figures S1–S4. For the first RSV season of the pandemic, the delay in RSV onset and peak was more pronounced in the northern temperate, whereas the delay in RSV offset was more pronounced in the southern temperate; no statistically significant findings were observed in the tropics where RSV activity was seasonal (Table 2). For the second RSV season of the pandemic, interestingly, we found that the RSV season ended earlier in the southern temperate, opposite from what was observed in the northern temperate; no statistically significant findings were observed in the tropics (Table 2).

**TABLE 2:** Comparison of RSV seasonality between 2009 influenza pandemic and inter‐pandemic periods, by latitude

Period	Onset		Peak		Onset‐peak interval		Offset		Duration	
	*N* of seasons	Difference in months	*N* of seasons	Difference in months	*N* of seasons	Difference in months	*N* of seasons	Difference in months	*N* of seasons	Difference in months
Pandemic (1st season) versus pre/post‐pandemic^a^										
Northern temperate	143	0.82 (0.61, 1.04)	168	0.41 (0.21, 0.62)	136	−0.38 (−0.66, −0.10)	139	−0.16 (−0.35, 0.03)	139	−0.97 (−1.27, −0.68)
Tropics	36	0.26 (−0.16, 0.69)	36	0.61 (−0.08, 1.31)	36	0.35 (−0.22, 0.92)	36	0.36 (−0.36, 1.08)	36	0.10 (−0.54, 0.74)
Southern temperate	59	0.18 (0.01, 0.34)	41	−0.36 (−0.75, 0.02)	41	−0.54 (−0.90, −0.19)	59	0.25 (0.01, 0.49)	59	0.05(−0.24, 0.34)
Pandemic (2nd season) versus pre/post‐pandemic^a^										
Northern temperate	127	0.37 (0.21, 0.53)	152	0.47 (0.28, 0.66)	120	−0.10 (−0.29, 0.08)	123	0.22 (0.06, 0.39)	123	−0.16 (−0.37, 0.05)
Tropics	16	0.73 (−0.09, 1.54)	16	−0.44 (−2.14, 1.27)	16	−1.16 (−2.78, 0.46)	16	0.38 (−0.44, 1.20)	16	−0.35 (−0.92, 0.23)
Southern temperate	59	−0.12 (−0.34, 0.10)	41	−0.26 (−0.53, 0.02)	41	−0.31 (−0.51, −0.12)	59	−0.99 (−1.38, −0.61)	59	−0.89 (−1.24, −0.55)

^a^ Pandemic (1st season) is defined as the first RSV season since April 2009. Pandemic (2nd season) is defined as the second RSV season since April 2009.

**FIGURE 3:**
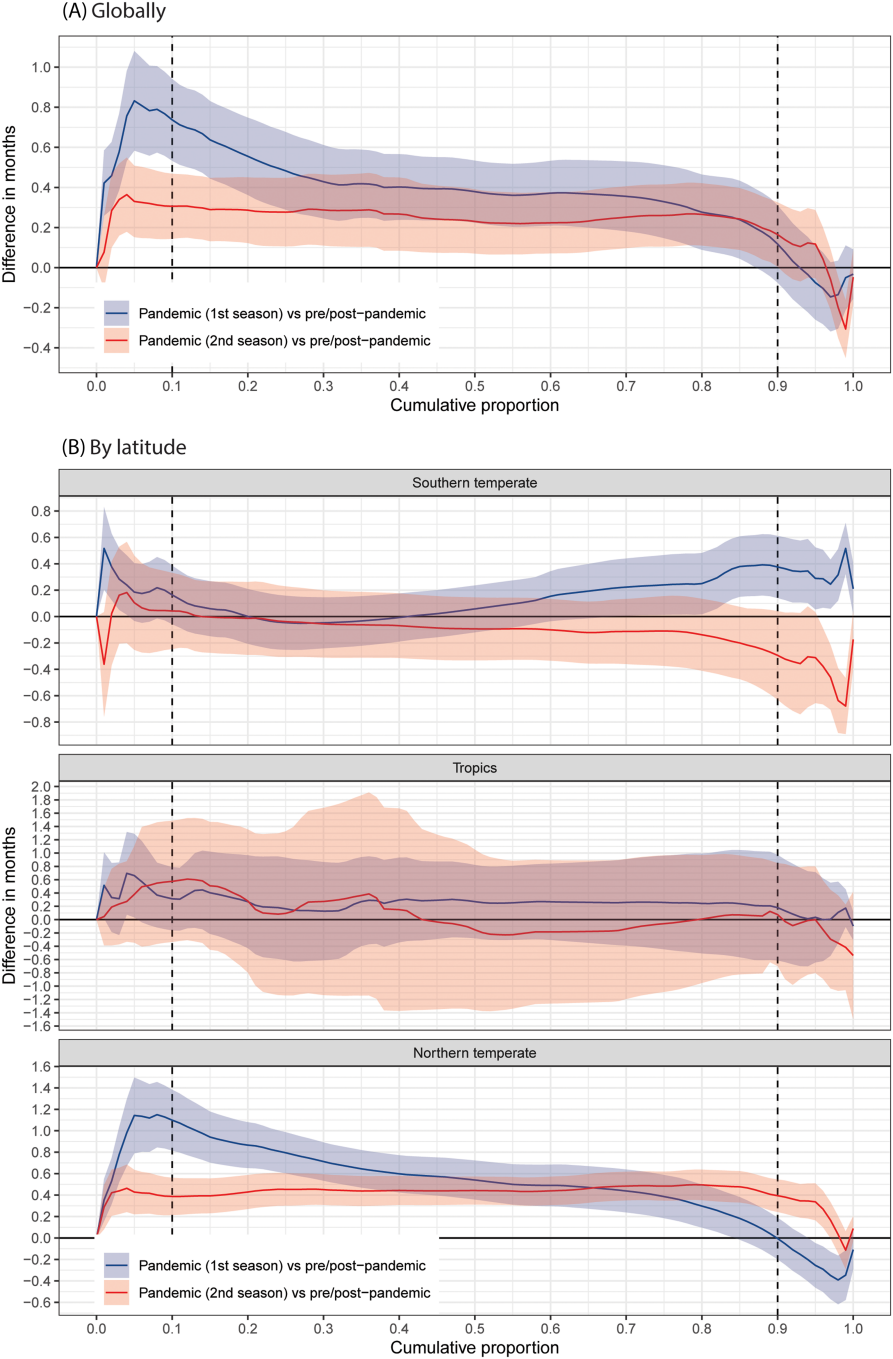
Comparison of time required to reach the same level of annual cumulative proportion of RSV between influenza pandemic and inter‐pandemic periods, globally (A) and by latitude (B). A positive difference indicates delayed activity of RSV. Reference is pre/post‐pandemic period. Pandemic (1st season) is defined as the first RSV season since April 2009. Pandemic (2nd season) is defined as the second RSV season since April 2009

The results of our exploratory analysis using only the RSV seasonality data confirmed the regional variations observed above but provided more details (Figure 3): in the northern temperate, the delay in observing the same level of cumulative RSV activity was most pronounced at the beginning of both the first and second RSV seasons since the pandemic, but the delay became less pronounced over the course of the season, and there was almost no delay at the season offset; in the southern temperate, a pronounced delay (0.38 months, 95% CI: 0.14, 0.61) was observed around the offset for the first RSV season and an advanced RSV epidemic (−0.29 months, 95% CI: −0.63, 0.04) was observed around the offset for the second RSV season, though not being statistically significant.

## 4 DISCUSSION

In this study, we highlighted a globally averaged delay of 0.58 months and a maximum delay of 2.5 months in the onset of the first RSV season in the influenza pandemic compared with the inter‐pandemic period. The delayed onset was most pronounced in the northern temperate, followed by the southern temperate, and was least pronounced in the tropics. The second RSV season was impacted to a lesser extent, and the third RSV season was not impacted.

Our findings suggest the presence of viral interference and potentially have important implications for preparedness for RSV season in the ongoing COVID‐19 pandemic, although there are differences worth noting between the 2009 influenza pandemic and the COVID‐19 pandemic. First, the viral pathogens causing the pandemic are different (i.e. influenza H1N1pdm vs. SARS‐CoV‐2) and viral interference effects could differ by viruses pair.^16^ Second, unlike during the COVID‐19 pandemic, no extensive non‐pharmaceutical interventions such as nationwide lockdowns were implemented during the influenza pandemic, although certain restrictions such as school closures and international travel limits were introduced by some countries.^17^ This could help explain why the RSV onset was delayed by several months in some countries in the southern hemisphere in 2020,^18^, ^19^ rather than a few weeks in the 2009 influenza pandemic as shown in our study. We also observed a shorter interval between RSV onset and peak in both the first and second seasons after the 2009 influenza pandemic, which possibly indicates that the post‐COVID‐19 RSV season might reach its peak earlier once it starts. Interestingly, compared with RSV, the circulation of human rhinovirus was less impacted by either the 2009 influenza pandemic^9^, ^10^ or the ongoing COVID‐19 pandemic,^20^, ^21^ which in turn supported the role of viral interference in the delayed RSV season although the typical age profile of infections differed among influenza, RSV, rhinovirus and SARS‐CoV‐2. In the present study, we found that the influenza pandemic affected the second RSV season to a lesser extent and that RSV season tended to revert to the pre‐pandemic state. This could provide clues for the timing of the second RSV season after the resumption of RSV activity in the COVID‐19 pandemic if no further major non‐pharmaceutical interventions are implemented.

Our findings also highlighted the regional variations in the effect of the influenza pandemic on RSV seasonality. We believe these variations could be explained by the timing of the emergence of the pandemic relative to the timing of the local RSV season. For example, we found that in the southern temperate, the onset of the RSV season was less affected compared with the offset of the RSV season. This was because RSV season in the southern temperate usually began in May and June, which were the months when the influenza pandemic just started in 2009. By comparison, we found that the onset of the RSV season was more affected compared with the offset of the RSV season in the northern temperate. This was because RSV season usually began in November and December in the northern temperate, by which time the influenza pandemic had already unfolded. For the tropics, however, we did not observe any statistically significant differences between RSV seasons in the pandemic and in the inter‐pandemic period, although the point estimates indicated that the RSV season might be delayed. This could be due to the lack of statistical power because the majority of the data were from temperate regions. This could also be due to the fact that the timing of RSV season was more varied in the tropics than the temperate regions.^3^

Our study has several strengths. First, we went beyond published literatures that reported the impact of the influenza pandemic on RSV seasonality (as summarised in Table S1), by compiling both RSV seasonality results and RSV seasonality data from various sources. This allowed us to analyse the best available data and helps reduce publication bias that tended to favour statistically significant results. Second, while focusing on RSV onset as the main outcome measure, we conducted several exploratory analyses to assess other measures of RSV season, such as peak, onset‐peak interval, offset and duration, as well as a novel measure that assessed the time length required to reach different levels of cumulative RSV activity. Third, we conducted several sets of subgroup analyses, for example, by latitude, to gain more insights into the regional‐specific impact of the influenza pandemic on RSV seasonality. However, we do acknowledge some limitations of our study. First, the underlying methodology of our compiled data varied greatly; nonetheless, because the comparisons between the pandemic and inter‐pandemic periods were made only within each site (i.e. site‐wise comparison), we do not expect our results to be affected substantially. Second, we were unable to assess any age‐specific effects of the influenza pandemic on RSV seasonality due to the lack of age‐disaggregated data. Third, we acknowledge that testing practice and method in each study site could change substantially over time, especially during and after the 2009 influenza pandemic; nonetheless, our subgroup analysis excluding the pre‐pandemic period showed similar results. Fourth, most of the study sites (31/48, 65%) were from northern hemisphere, and as a result, the global overall results were largely represented by northern hemisphere; in particular, we might lack the statistical power to differentiate the true impact of the influenza pandemic from the typical variations in the timing of RSV season for the tropics and southern hemisphere. Fifth, we were unable to compare the amplitude of RSV peak across different seasons due to the perceived change over time in the testing capacity. A recent modelling study by Baker and colleagues^22^ predicts that non‐pharmaceutical interventions could lead to larger future outbreaks in the second RSV season after the COVID‐19 pandemic (i.e. the year 2021), although that study did not account for viral interference in the model. Lastly, our results should be interpreted in the context of the differences, as noted above, between the 2009 influenza pandemic and the ongoing COVID‐19 pandemic.

RSV seasonality information is important for both public health services planning and timely administration of RSV prophylaxis. The experience from the 2009 influenza pandemic could potentially help prepare for the upcoming RSV season during the ongoing COVID‐19 pandemic and any possible future pandemics. RSV surveillance across multiple sites globally was interrupted during the COVID‐19 pandemic, and it is essential to resume these surveillance activities to understand the full profile of RSV epidemiology in the post‐COVID‐19 era.

## AUTHOR CONTRIBUTIONS

**You Li:** Conceptualization; data curation; formal analysis; methodology; supervision; visualization. **Xin Wang:** Data curation; formal analysis. **Takondwa Msosa:** Data curation. **Femke de Wit:** Data curation. **Jayne Murdock:** Data curation. **Harish Nair:** Conceptualization; supervision.

## CONFLICT OF INTERESTS

YL reports grants from WHO and Wellcome Trust, outside the submitted work. HN reports grants from the Innovative Medicines Initiative, WHO, and the National Institute for Health Research; personal fees from the Bill & Melinda Gates Foundation, Janssen, ReViral and AbbVie; and grants and personal fees from Sanofi and the Foundation for Influenza Epidemiology, outside the submitted work. All other authors declare no conflicts of interest.

## Supporting information


**Table S1.** Summary of studies reporting the impact of the 2009 influenza pandemic on RSV seasonality
**Table S2.** Summary of included data from literature review, online datasets and previously published data on global RSV seasonality
**Table S3.** Comparison of RSV seasonality between 2009 influenza pandemic and inter‐pandemic periods, using seasonality‐results‐preferred approach (sensitivity analysis 1)
**Table S4.** Comparison of RSV seasonality between 2009 influenza pandemic and inter‐pandemic periods, excluding studies with one or more C‐rating in the quality assessment (sensitivity analysis 2)
**Table S5.** Comparison of RSV seasonality between 2009 influenza pandemic and inter‐pandemic periods, excluding studies with less than five RSV seasons (sensitivity analysis 3)
**Figure S1.** Comparison of RSV peak between 2009 influenza pandemic and inter‐pandemic periods by study site
**Figure S2.** Comparison of RSV onset‐to‐peak interval between 2009 influenza pandemic and inter‐pandemic periods by study site
**Figure S3.** Comparison of RSV offset between 2009 influenza pandemic and inter‐pandemic periods by study site
**Figure S4.** Comparison of RSV duration between 2009 influenza pandemic and inter‐pandemic periods by study site

## Data Availability

The study data are available from the corresponding author upon reasonable request.
