# Supplementary material for: The impact of the 2009 influenza pandemic on the seasonality of human respiratory syncytial virus: A systematic analysis
Source: Influenza Other Respir Viruses. 2021 Jul 4;15(6):804–12. doi: 10.1111/irv.12884 (PMC8542946; doi:10.1111/irv.12884)
Supplement: Supplementary file 1 — Table S1. Summary of studies reporting the impact of the 2009 influenza pandemic on RSV seasonality Table S2. Summary of included data from literature review, online datasets and previously published data on global RSV seasonality Table S3. Comparison of RSV seasonality between 2009 influenza pandemic and inter‐pandemic periods, using seasonality‐results‐preferred approach (sensitivity analysis 1) Table S4. Comparison of RSV seasonality between 2009 influenza pandemic and inter‐pandemic periods, excluding studies with one or more C‐rating in the quality assessment (sensitivity analysis 2) Table S5. Comparison of RSV seasonality between 2009 influenza pandemic and inter‐pandemic periods, excluding studies with less than five RSV seasons (sensitivity analysis 3) Figure S1. Comparison of RSV peak between 2009 influenza pandemic and inter‐pandemic periods by study site Figure S2. Comparison of RSV onset‐to‐peak interval between 2009 influenza pandemic and inter‐pandemic periods by study site Figure S3. Comparison of RSV offset between 2009 influenza pandemic and inter‐pandemic periods by study site Figure S4. Comparison of RSV duration between 2009 influenza pandemic and inter‐pandemic periods by study site [file IRV-15-804-s001.docx]

Contents

[Text S1. Search strategy 2](#_Toc69221876)

[MEDLINE (Ovid) 2](#_Toc69221877)

[EMBASE (Ovid) 2](#_Toc69221878)

[Global Health (Ovid) 2](#_Toc69221879)

[Text S2. Quality assessment questionnaire 3](#_Toc69221880)

[Table S1. Summary of studies reporting the impact of the 2009 influenza pandemic on RSV seasonality 4](#_Toc69221881)

[Table S2. Summary of included data from literature review, online datasets and previously published data on global RSV seasonality 5](#_Toc69221882)

[Table S3. Comparison of RSV seasonality between 2009 influenza pandemic and inter-pandemic periods, using seasonality-results-preferred approach (sensitivity analysis 1) 8](#_Toc69221883)

[Table S4. Comparison of RSV seasonality between 2009 influenza pandemic and inter-pandemic periods, excluding studies with one or more C-rating in the quality assessment (sensitivity analysis 2) 9](#_Toc69221884)

[Table S5. Comparison of RSV seasonality between 2009 influenza pandemic and inter-pandemic periods, excluding studies with less than five RSV seasons (sensitivity analysis 3) 10](#_Toc69221885)

[Figure S1. Comparison of RSV peak between 2009 influenza pandemic and inter-pandemic periods by study site 11](#_Toc69221886)

[Figure S2. Comparison of RSV onset-to-peak interval between 2009 influenza pandemic and inter-pandemic periods by study site 12](#_Toc69221887)

[Figure S3. Comparison of RSV offset between 2009 influenza pandemic and inter-pandemic periods by study site 13](#_Toc69221888)

[Figure S4. Comparison of RSV duration between 2009 influenza pandemic and inter-pandemic periods by study site 14](#_Toc69221889)

[PRISMA checklist 15](#_Toc69221890)

[References 17](#_Toc69221891)

# Text S1. Search strategy

## MEDLINE (Ovid)

1. bronchiolitis.mp. or exp Bronchiolitis/

2. *Respiratory Syncytial Viruses/ or *Respiratory Syncytial Virus Infections/ or respiratory syncytial.mp. or *Respiratory Syncytial Virus, Human/

3. 1 or 2

4. Seasons/ or season*.mp.

5. temporal.mp. or exp Spatio-Temporal Analysis/

6. periodic*.mp. or exp Periodicity/

7. surveillance.ti,ab. or exp Population Surveillance/

8. 4 or 5 or 6 or 7

9. ep.fs.

10. 3 and 8 and 9

11. limit 10 to yr="2009 - 2020"

**1181** records retrieved on 3^rd^ March 2021.

## EMBASE (Ovid)

1. respiratory syncytial virus.mp. or exp Human respiratory syncytial virus/

2. bronchiolitis.mp. or exp bronchiolitis/ or exp viral bronchiolitis/

3. 1 or 2

4. exp season/ or season*.mp. or exp seasonal variation/

5. exp Population Surveillancel/ or surveillance.ti,ab.

6. periodic*.mp. or exp periodicity/

7. exp spatiotemporal analysis/ or temporal.mp.

8. 4 or 5 or 6 or 7

9. ep.fs.

10. 3 and 8 and 9

11. limit 10 to yr="2009 - 2020"

**1136** records retrieved on 3^rd^ March 2021.

## Global Health (Ovid)

1. bronchiolitis.mp. or exp bronchiolitis/

2. respiratory syncytial.mp. or Human respiratory syncytial virus.od.

3. 1 or 2

4. exp seasonal variation/ or exp seasonality/ or season*.mp.

5. exp surveillance/ or surveillance.mp. or exp sentinel surveillance/

6. temporal.mp. or temporal variation/

7. periodic*.mp. or exp periodicity/

8. 4 or 5 or 6 or 7

9. 3 and 8

10. limit 9 to yr="2009 - 2020"

**1445** records retrieved on 3^rd^ March 2021.

# Text S2. Quality assessment questionnaire

| **Q1. Were subjects included representative of the population in the study site?** | | |
| --- | --- | --- |
|  | **A. Yes. Very good representativeness.** | Subjects were all ages, without underlying medical conditions and not from a specific SES group (e.g. slum) |
|  | **B. Yes. Good representativeness.** | Subjects were not all ages, without underlying medical conditions and not from a specific SES group |
|  | **C. Yes. Likely to be representative with regard to seasonality of viruses** | Subjects were from very narrow age bands (e.g. neonatal), without underlying medical condition; OR subjects were from a specific SES group. |
|  | **D. No. Unlikely to be representative. (Should be excluded)** | Subjects with underlying medical conditions. |
| **Q2. Did the test method(s) and practice remain stable throughout study period?** | | |
|  | **A. Yes. Very stable.** | One or two test methods with stable test practice throughout study period. |
|  | **B. Yes. Stable.** | >2 test methods with stable test practice throughout study period. |
|  | **C. No, but changes were unlikely to affect seasonality results.** | >2 test methods with changes of test practice but should not affect seasonality results. |
|  | **D. No. Changes were unlikely to affect seasonality results. (Should be excluded)** | Changes of test methods or practice related to known seasonality. |
| **Q3. What was the quality of the timings of positive test results reported?** | | |
|  | **A. Very good.** | Timings of positive tests were reported as the date when specimens were taken (often found in prospective studies). |
|  | **B. Good.** | Timings of positive tests were taken from related diagnosis (often found in hospital databases). |
|  | **C. Fair.** | Timings of positive tests were reported as the date when specimens were received in labs (can be found in laboratory databases). |
|  | **D. Bad. (Should be excluded)** | Timings of positive tests could be significantly inaccurate and could affect seasonality results. |

SES = socioeconomic status.

# Table S1. Summary of studies reporting the impact of the 2009 influenza pandemic on RSV seasonality

| Study | Country and period | Main finding(s) |
| --- | --- | --- |
| Casalegno 2010^1^ | France; 2008–10 | - Delayed circulation of RSV in 2009–10 compared with 2008–09. |
| Grondahl 2014^2^ | Germany; 2009–11 | - RSV epidemics were several weeks later in 2009–10 than expected based on data for the previous 10 years but were as expected in 2010–11. |
| Hirsh 2014^3^ | Israel; 2005–12 | - RSV onset in 2009–10 was five weeks later than 2005–06 to 2008–09. - RSV onset in 2010–11 was the same as 2005–06 to 2008–09. |
| Mak 2012^4^ | Hong Kong, China; 2009–11 | - The usual RSV summer peak was not observed in both 2009 and 2010. - There was abnormal early rise of RSV activity in the winter of 2010. |
| Meningher 2014^5^ | Israel; 2007–12 | - RSV season was delayed in 2009–10. |
| Navarro-Mari 2012^6^ | Spain; 2009–10 | - The epidemic wave of respiratory syncytial virus during 2009–10 was similar to previous seasons. |
| Yang 2015^7^ | Hong Kong, China; 2004–13 | - Delayed RSV peak was observed in 2009–10. |
| Yang 2012^8^ | China; 2006–10 | - RSV season was delayed in 2009–10. |

RSV = respiratory syncytial virus.

# Table S2. Summary of included data from literature review, online datasets and previously published data on global RSV seasonality

| Data source | Location and country | Period | Subject age and case definition | Specimen(s) and testing method(s) | Seasonality data (weekly, monthly) | Seasonality results (on/offset, duration, peak) | Seasonality methods | Quality assessment | | |
| --- | --- | --- | --- | --- | --- | --- | --- | --- | --- | --- |
|  |  |  |  |  |  |  |  | QA1 | QA2 | QA3 |
| Published literatures | | | | | | | | | | |
| Ali 2017^9^ | Karachi, Pakistan | 2009/08–2012/07 | <5y, ARI | throat swabs, PCR | Monthly | No | NA | B | A | A |
| Althouse 2018^10^ | Nha Trang City, Vietnam | 2007/02–2012/04 | <5y, ARI | nasopharyngeal specimens, PCR | Monthly | No | NA | B | A | A |
| Balmaks 2014^11^ | Riga, Latvia | 2009/07–2012/06 | <2y, LRI | NPA, PCR | Monthly | No | NA | B | A | A |
| Bruden 2015^12^ | Yukon-Kuskokwim Delta, USA | 1994/07–2012/06 | <3y, LRI | NPA, RADT | Monthly | Onset, offset, duration and peak | “10% positivity threshold” method ^13^ | B | A | A |
| Buchan 2019^14^ | Ontario, Canada | 2009/05–2014/08 | <5y, NA | multiple, multiple | Monthly | No | NA | B | A | A |
| Callahan 2020^15^ | Intermountain west region, USA | 2005/07–2018/06 | all, NA | multiple, multiple | No | Peak | NA | A | B | C |
| Cui 2016^16^ | Eastern China, China | 2009/01–2013/12 | all, ALRI | NA, PCR | Monthly | No | NA | B | A | A |
| Cui 2013^17^ | Beijing, China | 2007/07–2012/06 | children, ARI | NPA, DFA or IFA | Monthly | No | NA | B | A | A |
| Dong 2015^18^ | Wenzhou, Taizhou and Lishui, China | 2009/07–2014/06 | <5y, LRTI | nasopharyngeal secretions, DFA | Monthly | No | NA | B | C | B |
| Feng 2014^19^ | 22 provinces, China | 2009/01–2013/09 | all, ALRI | multiple, NA | Monthly | No | NA | A | A | A |
| Ferone 2014^20^ | São Paulo, Brazil | 2008/03–2011/08 | <2y, ALRI | NPA, PCR | Monthly | No | NA | C | A | A |
| Ferrero 2016^21^ | Buenos Aires, Argentina | 1995/01–2014/12 | children, NA | NA, NA | No | Onset, offset and duration | "top 60% threshold" method ^22^ | B | B | B |
| Gamba-Sanchez 2016^23^ | Bogota , Colombia | 2009/01–2013/12 | <3y, ALRI | NPS, RADT | Monthly | No | NA | B | A | B |
| Gentile 2019^24^ | Buenos Aires, Argentina | 2000/01–2017/12 | <18y, ALRI | nasopharyngeal specimens, IFA or PCR | Monthly | No | NA | B | A | A |
| Hamada 2014^25^ | Yachiyo, Japan | 2007/04–2012/03 | <2y, LRI | NPA, PCR | Monthly | No | NA | B | A | B |
| He 2014^26^ | Shenzhen, China | 2007/07–2010/06 | <14y, ARI | NPA, PCR | Monthly | No | NA | B | A | A |
| Hirsh 2014^3^ | Ramat Gan, Israel | 2005/10–2012/03 | all, respiratory illness | NPA or NPS, PCR | No | Onset | NA | A | A | A |
| Horton 2017^27^ | Nationwide, Egypt, Jordan and Oman | 2007/12–2014/02 | all, SARI | NPS or OPS, PCR | Monthly | No | NA | A | A | A |
| Kang 2013^28^ | Seoul, South Korea | 2007/09–2012/07 | children, NA | nasopharyngeal specimens, PCR | Monthly | No | NA | A | A | C |
| Kanou 2018^29^ | Nationwide, Japan | 2008/01–2015/12 | all, NA | multiple, multiple | Weekly | No | NA | A | A | A |
| Kim 2017^30^ | Cheonan, South Korea | 2006/12–2014/02 | <1y, respiratory illness | NPA, PCR | Monthly | No | NA | C | A | A |
| Liu 2014^31^ | Shanghai, China | 2009/08–2012/12 | children, respiratory illness | NPS, PCR | Monthly | No | NA | B | A | A |
| Mak 2012^4^ | Hong Kong, China | 2004/01–2011/07 | all, NA | multiple, culture | Monthly | No | NA | C | C | C |
| Midgley 2017^32^ | Four census regions nationwide, USA | 2009/07–2015/06 | all, NA | multiple, PCR | No | Onset, offset, duration and peak | “retrospective slope” method ^32^ | A | A | A |
| Naorat 2013^33^ | Sa Kaeo and Nakhon Phanom, Thailand | 2008/01–2011/12 | all, ALRI | NPS, PCR | Monthly | No | NA | B | A | A |
| Nyoka 2019^34^ | Dadaab, Kenya | 2007/09–2011/08 | all, ILI and SARI | NA, NA | Monthly | No | NA | C | C | C |
| Onozuka 2015^35^ | Fukuoka, Japan | 2006/01–2012/12 | <5y, respiratory illness | NPS or NPA, antigen test or PCR | Weekly | No | NA | A | A | B |
| Reeves 2017^36^ | England, UK | 2007/07–2012/06 | all, respiratory illness | multiple, multiple | Weekly | No | NA | A | A | A |
| Renko 2020^37^ | Nationwide, Finland | 1995/01–2018/12 | all, NA | multiple, multiple | No | Peak | NA | A | B | B |
| Ucakar 2013^38^ | Nationwide, Slovenia | 2006/10–2011/09 | all, respiratory illness | multiple, PCR | No | Onset, offset and duration | “10% positivity threshold” method ^13^ | B | A | A |
| Vos 2019^39^ | Nationwide, Netherlands | 2005/08–2017/07 | all, respiratory illness | multiple, multiple | Weekly | Onset, offset, duration and peak | “MEM” method ^40^ | A | A | A |
| Yu 2019^41^ | Beijing, China | 2007/07–2015/06 | <14y, pneumonia | NPA, PCR | Monthly | Onset, offset, duration and peak | “10% positivity threshold” method ^13^ | B | A | A |
| Online reports/datasets | | | | | | | | | | |
| FluWatch (Canada)^42^ | Nationwide, Canada | 2008/08–2019/08 | all, ILI | multiple, multiple | Weekly | No | NA | A | B | A |
| IASR (Japan)^43^ | Nationwide, Japan | 2000/01–2014/12 | all, ARI | multiple, multiple | Monthly | No | NA | A | B | A |
| ESR Virology Annual Report^44^ | Nationwide, New Zealand | 2000/01–2017/12 | all, NA | multiple, multiple | Monthly | No | NA | A | B | A |
| Data published previously on global RSV seasonality^45^ | | | | | | | | | | |
| Clara | Santa Ana, El Salvador | 2008/01–2012/12 | <5y, ALRI | NPS, IFA | Monthly | No | NA | B | A | A |
| Homaira | New South Wales, Australia | 2001/01–2010/12 | all, respiratory illness | multiple, multiple | Weekly | No | NA | A | B | B |
| McCraken | Santa Rosa, Guatemala | 2008/01–2013/12 | <5y, ALRI | NPS and OPS, PCR | Monthly | No | NA | B | A | A |
| McCraken | Quetzaltenango, Guatemala | 2009/02–2013/12 | <5y, ALRI | NPS and OPS, PCR | Monthly | No | NA | B | A | A |
| Nokes | Kilifi, Kenya | 2002/01–2010/12 | <5y, ALRI | NPS or OPS, DFA | Monthly | No | NA | B | A | A |
| Noyola | San Luis Potosí, Mexico | 2003/07–2014/12 | <5y, ALRI | NPA, immuno-fluorescence or PCR | Monthly | No | NA | A | A | A |
| Simoes | State of Colorado, USA | 2008/01–2013/06 | <5y, ALRI | nasal wash, PCR | Monthly | No | NA | B | A | A |
| Li† | Scotland, UK | 2009/08–2018/07 | all, respiratory illness | multiple, multiple | Weekly | No | NA | A | A | A |

†Included from the doctoral thesis of You Li^46^.

ALRI = acute lower respiratory infection; ARI = acute respiratory infection; DFA = direct fluorescent assay; IFA = indirect fluorescent assay; ILI = influenza-like illness; LRTI = lower respiratory tract infection; NA = not available; NPA = nasopharyngeal aspirate; NPS = nasopharyngeal swab; OPS = oropharyngeal swab; PCR = polymerase chain reaction; RADT = rapid antigen detection test; SARI = severe acute respiratory infection.

# Table S3. Comparison of RSV seasonality between 2009 influenza pandemic and inter-pandemic periods, using seasonality-results-preferred approach (sensitivity analysis 1)

| Period | **Onset** | | **Peak** | | **Onset-peak interval** | | **Offset** | | **Duration** | |
| --- | --- | --- | --- | --- | --- | --- | --- | --- | --- | --- |
|  | N of seasons | Difference in months | N of seasons | Difference in months | N of seasons | Difference in months | N of seasons | Difference in months | N of seasons | Difference in months |
| Pandemic (1^st^ season) vs pre/post-pandemic† | **238** | **0.65 (0.49, 0.81)** | **245** | **0.29 (0.10, 0.48)** | **213** | **–0.39 (–0.63, –0.16)** | **234** | **0.05 (–0.11, 0.21)** | **234** | **–0.59 (–0.81, –0.38)** |
| Pandemic (1^st^ season) vs  Pre-pandemic† | 128 | 0.75 (0.51, 0.98) | 127 | 0.22 (–0.08, 0.52) | 108 | –0.61 (–1.01, –0.21) | 125 | 0.01 (–0.21, 0.23) | 125 | –0.73 (–1.07, –0.40) |
| Pandemic (1^st^ season) vs  Post-pandemic† | 110 | 0.53 (0.32, 0.74) | 118 | 0.36 (0.14, 0.59) | 105 | –0.17 (–0.41, 0.07) | 109 | 0.10 (–0.14, 0.34) | 109 | –0.43 (–0.68, –0.18) |
| Pandemic (2^nd^ season) vs  pre/post-pandemic† | **202** | **0.28 (0.14, 0.42)** | **209** | **0.26 (0.06, 0.47)** | **177** | **–0.27 (–0.47, –0.07)** | **198** | **–0.16 (–0.34, 0.02)** | **198** | **–0.46 (–0.63, –0.29)** |
| Pandemic (2^nd^ season) vs  Pre-pandemic† | 97 | 0.001 (–0.17, 0.17) | 96 | 0.21 (–0.13, 0.54) | 77 | –0.16 (–0.53, 0.21) | 94 | –0.30 (–0.61, 0.01) | 94 | –0.32 (–0.60, –0.03) |
| Pandemic (2^nd^ season) vs  Post-pandemic† | 105 | 0.54 (0.34, 0.75) | 113 | 0.31 (0.07, 0.56) | 100 | –0.35 (–0.56, –0.14) | 104 | –0.04 (–0.24, 0.17) | 104 | –0.59 (–0.77, –0.40) |

†Reference.

If a study reported both seasonality results (i.e. on/offset, peak and duration) and activity data (e.g. weekly/monthly counts), we preferred to use seasonality results for the analysis, which was different from the main analysis that preferred to use activity data.

Pandemic (1st season) is defined as the first RSV season since April 2009. Pandemic (2nd season) is defined as the second RSV season since April 2009.

# Table S4. Comparison of RSV seasonality between 2009 influenza pandemic and inter-pandemic periods, excluding studies with one or more C-rating in the quality assessment (sensitivity analysis 2)

| Period | **Onset** | | **Peak** | | **Onset–peak interval** | | **Offset** | | **Duration** | |
| --- | --- | --- | --- | --- | --- | --- | --- | --- | --- | --- |
|  | N of seasons | Difference in months | N of seasons | Difference in months | N of seasons | Difference in months | N of seasons | Difference in months | N of seasons | Difference in months |
| Pandemic (1^st^ season) vs pre/post-pandemic† | 221 | 0.60 (0.44, 0.76) | 217 | 0.33 (0.14, 0.53) | 196 | –0.30 (–0.52, –0.08) | 217 | 0.04 (–0.13, 0.21) | 217 | –0.56 (–0.79, –0.32) |
| Pandemic (1^st^ season) vs  Pre-pandemic† | 117 | 0.68 (0.43, 0.93) | 111 | 0.28 (–0.02, 0.58) | 97 | –0.47 (–0.82, –0.11) | 114 | –0.004 (–0.23, 0.23) | 114 | –0.68 (–1.06, –0.31) |
| Pandemic (1^st^ season) vs  Post-pandemic† | 104 | 0.51 (0.30, 0.72) | 106 | 0.39 (0.14, 0.63) | 99 | –0.14 (–0.38, 0.11) | 103 | 0.09 (–0.15, 0.34) | 103 | –0.41 (–0.67, –0.16) |
| Pandemic (2^nd^ season) vs  pre/post-pandemic† | 185 | 0.21 (0.07, 0.36) | 181 | 0.34 (0.16, 0.53) | 160 | –0.13 (–0.27, 0.013) | 181 | –0.19 (–0.38, –0.002) | 181 | –0.42 (–0.60, –0.24) |
| Pandemic (2^nd^ season) vs  Pre-pandemic† | 86 | –0.08 (–0.25, 0.09) | 80 | 0.38 (0.12, 0.63) | 66 | 0.09 (–0.06, 0.24) | 83 | –0.39 (–0.71, –0.06) | 83 | –0.32 (–0.62, –0.02) |
| Pandemic (2^nd^ season) vs  Post-pandemic† | 99 | 0.47 (0.25, 0.68) | 101 | 0.32 (0.05, 0.58) | 94 | –0.29 (–0.50, –0.07) | 98 | –0.03 (–0.25, 0.19) | 98 | –0.50 (–0.72, –0.28) |

†Reference.

Pandemic (1^st^ season) is defined as the first RSV season since April 2009. Pandemic (2^nd^ season) is defined as the second RSV season since April 2009.

# Table S5. Comparison of RSV seasonality between 2009 influenza pandemic and inter-pandemic periods, excluding studies with less than five RSV seasons (sensitivity analysis 3)

| Period | **Onset** | | **Peak** | | **Onset–peak interval** | | **Offset** | | **Duration** | |
| --- | --- | --- | --- | --- | --- | --- | --- | --- | --- | --- |
|  | N of seasons | Difference in months | N of seasons | Difference in months | N of seasons | Difference in months | N of seasons | Difference in months | N of seasons | Difference in months |
| Pandemic (1^st^ season) vs pre/post-pandemic† | 213 | 0.62 (0.47, 0.78) | 220 | 0.29 (0.10, 0.47) | 188 | –0.37 (–0.59, –0.15) | 209 | –0.01 (–0.18, 0.15) | 209 | –0.64 (–0.87, –0.40) |
| Pandemic (1^st^ season) vs  Pre-pandemic† | 116 | 0.69 (0.44, 0.93) | 115 | 0.17 (–0.15, 0.48) | 96 | –0.61 (–0.98, –0.24) | 113 | –0.19 (–0.42, 0.04) | 113 | –0.88 (–1.24, –0.51) |
| Pandemic (1^st^ season) vs  Post-pandemic† | 97 | 0.55 (0.36, 0.74) | 105 | 0.42 (0.25, 0.59) | 92 | –0.13 (–0.34, 0.09) | 96 | 0.20 (–0.04, 0.43) | 96 | –0.35 (–0.61, –0.09) |
| Pandemic (2^nd^ season) vs  pre/post-pandemic† | 183 | 0.20 (0.07, 0.34) | 190 | 0.23 (0.05, 0.42) | 158 | –0.25 (–0.44, –0.05) | 179 | –0.18 (–0.35, –0.01) | 179 | –0.40 (–0.56, –0.23) |
| Pandemic (2^nd^ season) vs  Pre-pandemic† | 89 | –0.02 (–0.19, 0.15) | 88 | 0.13 (–0.23, 0.48) | 69 | –0.28 (–0.67, 0.11) | 86 | –0.43 (–0.73, –0.12) | 86 | –0.42 (–0.70, –0.14) |
| Pandemic (2^nd^ season) vs  Post-pandemic† | 94 | 0.42 (0.23, 0.60) | 102 | 0.32 (0.16, 0.49) | 89 | –0.22 (–0.39, –0.05) | 93 | 0.05 (–0.11, 0.21) | 93 | –0.37 (–0.56, –0.18) |

†Reference.

Pandemic (1^st^ season) is defined as the first RSV season since April 2009. Pandemic (2^nd^ season) is defined as the second RSV season since April 2009.

# Figure S1. Comparison of RSV peak between 2009 influenza pandemic and inter-pandemic periods by study site


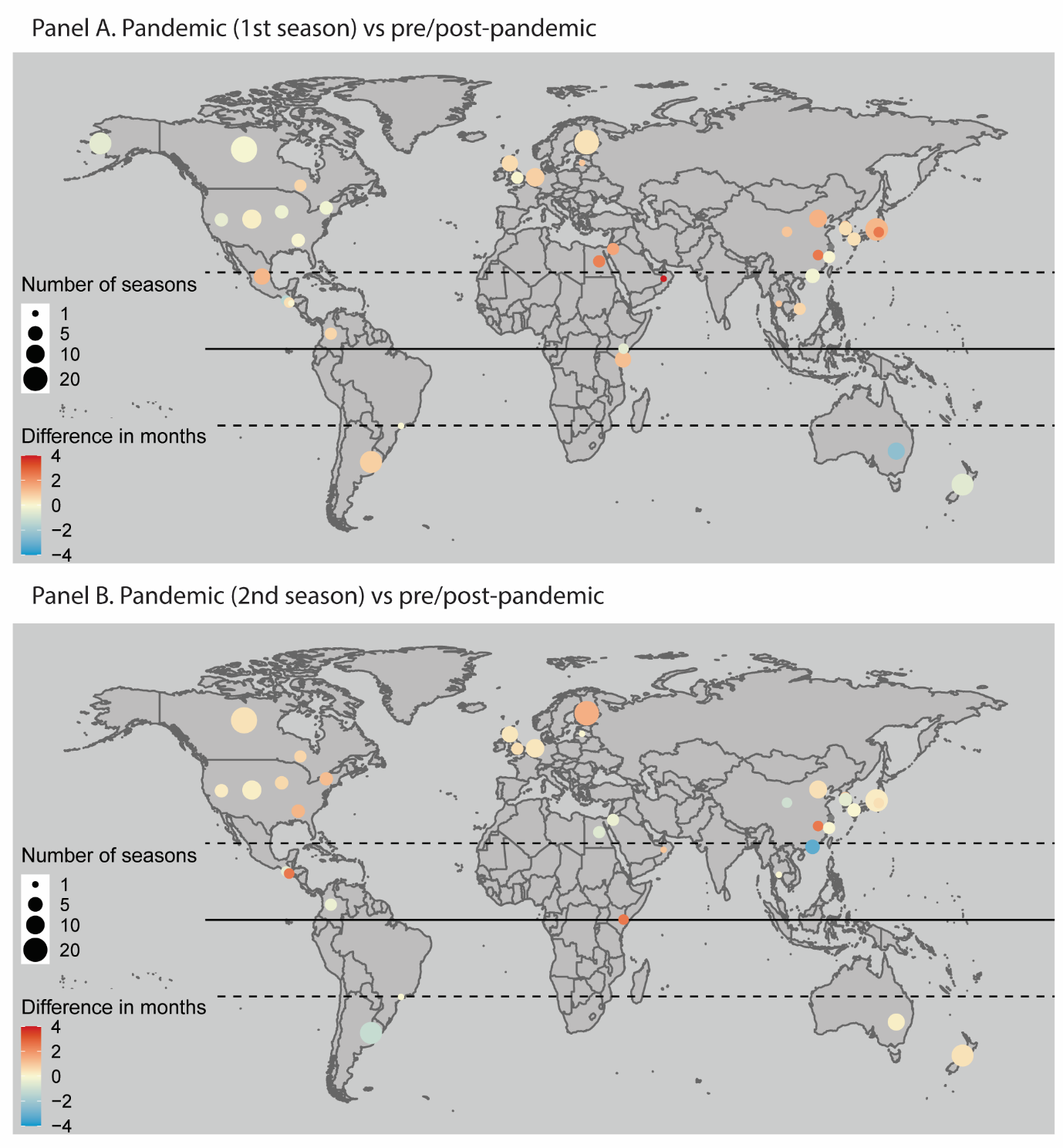


Reference is pre/post-pandemic period. Pandemic (1^st^ season) is defined as the first RSV season since April 2009. Pandemic (2^nd^ season) is defined as the second RSV season since April 2009.

# Figure S2. Comparison of RSV onset-to-peak interval between 2009 influenza pandemic and inter-pandemic periods by study site


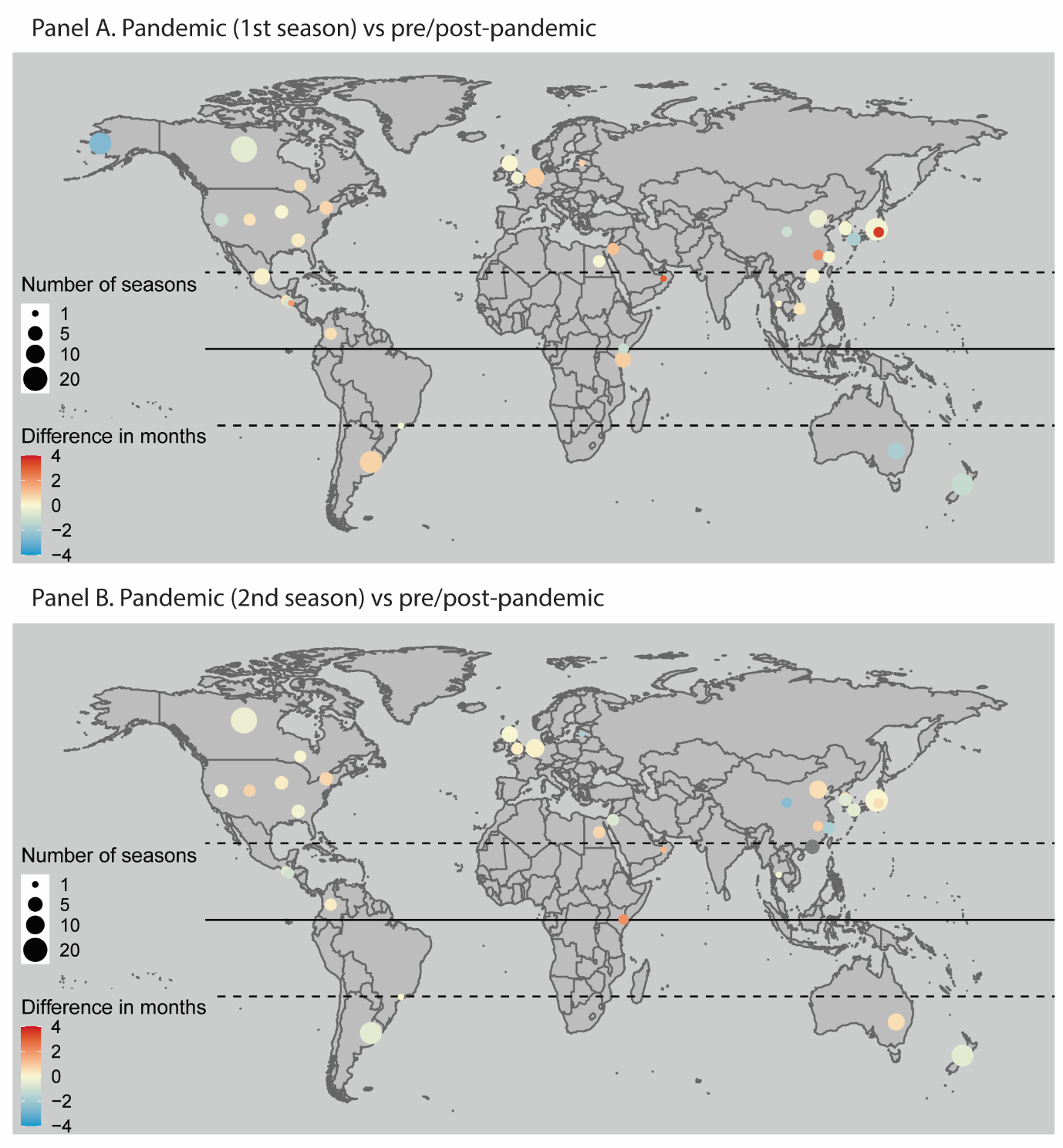


Reference is pre/post-pandemic period. Pandemic (1^st^ season) is defined as the first RSV season since April 2009. Pandemic (2^nd^ season) is defined as the second RSV season since April 2009.

# Figure S3. Comparison of RSV offset between 2009 influenza pandemic and inter-pandemic periods by study site


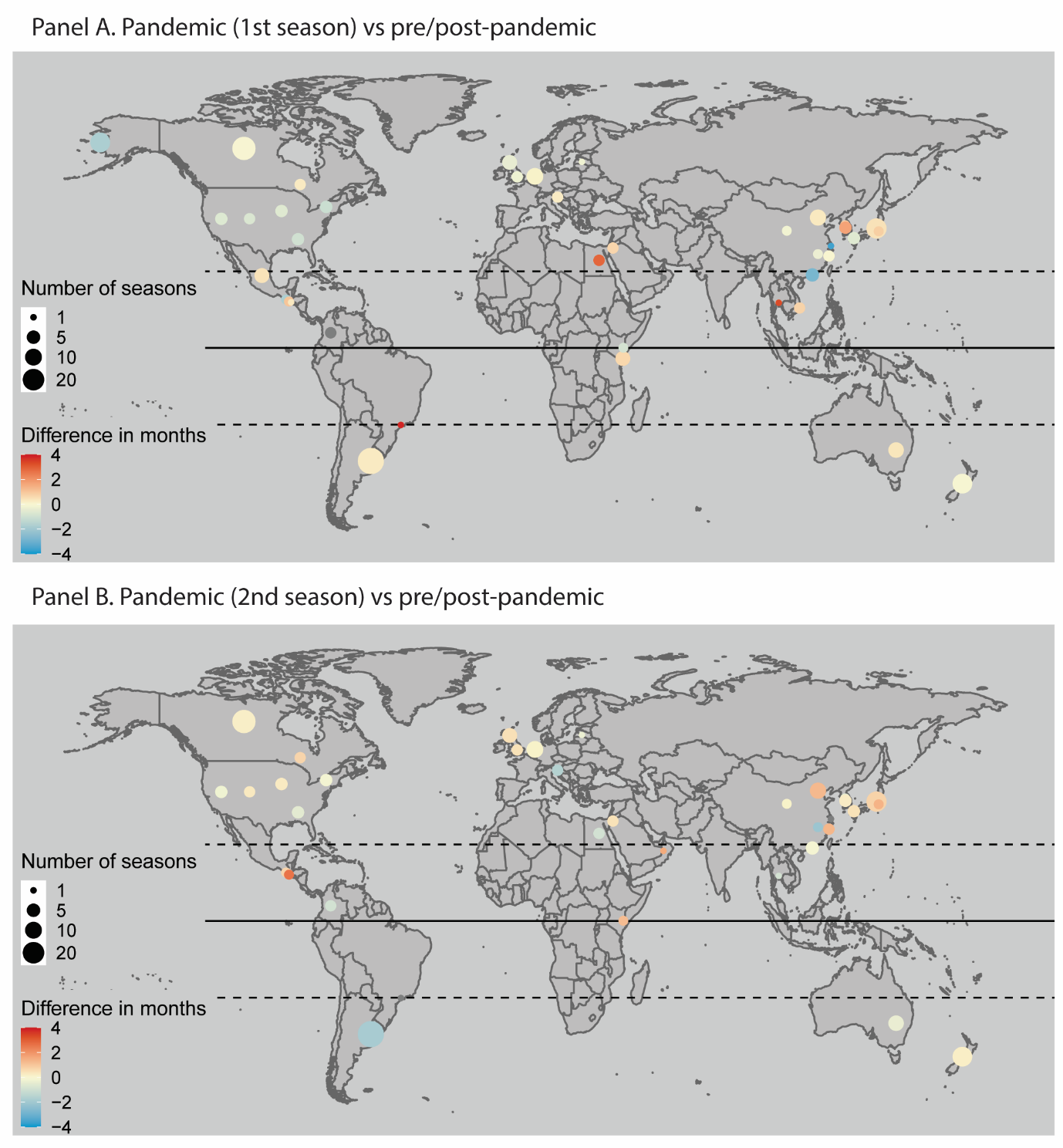


Reference is pre/post-pandemic period. Pandemic (1^st^ season) is defined as the first RSV season since April 2009. Pandemic (2^nd^ season) is defined as the second RSV season since April 2009.

# Figure S4. Comparison of RSV duration between 2009 influenza pandemic and inter-pandemic periods by study site


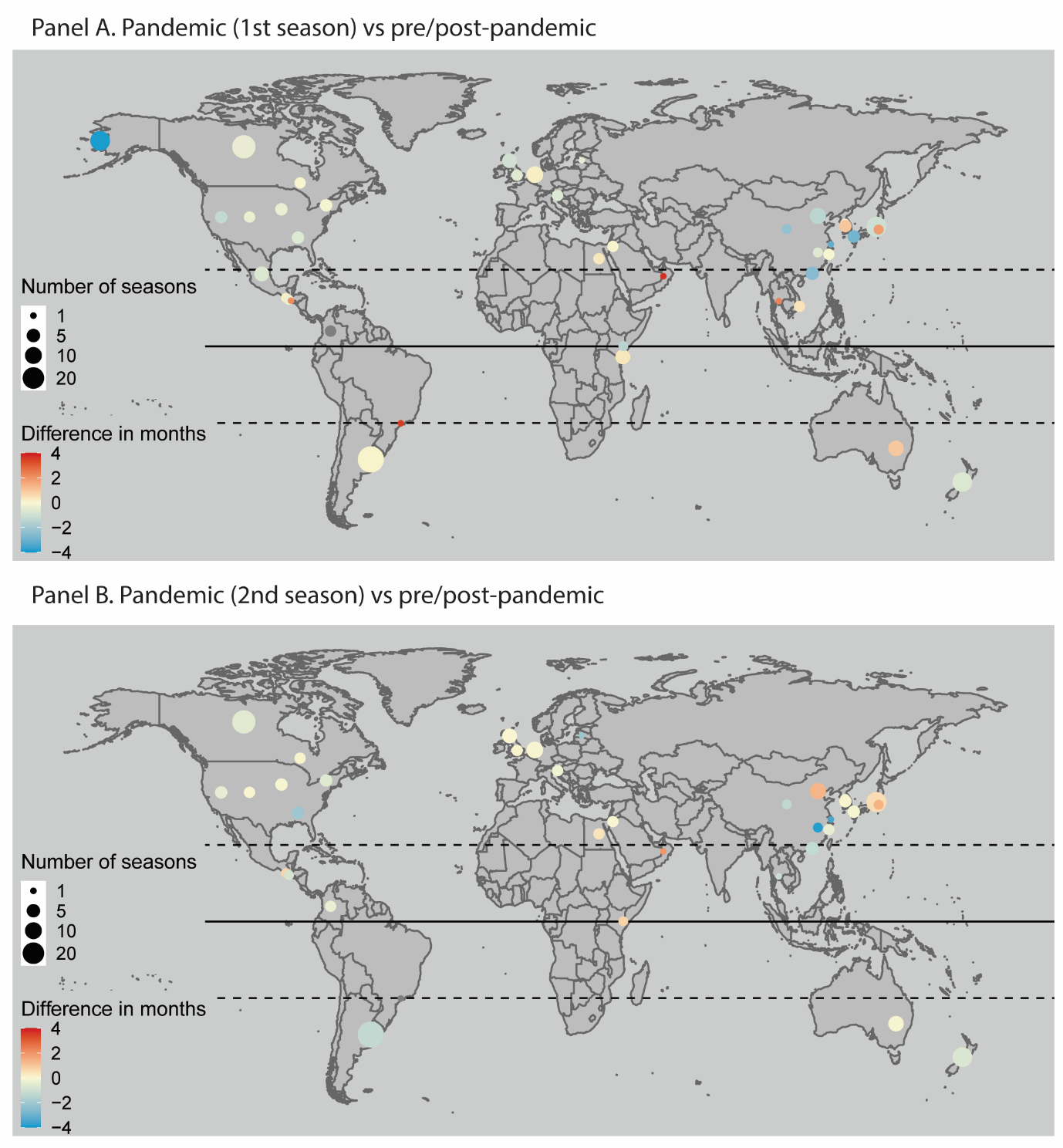


Reference is pre/post-pandemic period. Pandemic (1^st^ season) is defined as the first RSV season since April 2009. Pandemic (2^nd^ season) is defined as the second RSV season since April 2009.

# PRISMA checklist

| **Section/topic** | **#** | **Checklist item** | **Reported on page #** |
| --- | --- | --- | --- |
| **TITLE** | | |  |
| Title | 1 | Identify the report as a systematic review, meta-analysis, or both. | 1 |
| **ABSTRACT** | | |  |
| Structured summary | 2 | Provide a structured summary including, as applicable: background; objectives; data sources; study eligibility criteria, participants, and interventions; study appraisal and synthesis methods; results; limitations; conclusions and implications of key findings; systematic review registration number. | 2 |
| **INTRODUCTION** | | |  |
| Rationale | 3 | Describe the rationale for the review in the context of what is already known. | 3 |
| Objectives | 4 | Provide an explicit statement of questions being addressed with reference to participants, interventions, comparisons, outcomes, and study design (PICOS). | 3 |
| **METHODS** | | |  |
| Protocol and registration | 5 | Indicate if a review protocol exists, if and where it can be accessed (e.g., Web address), and, if available, provide registration information including registration number. | 4 |
| Eligibility criteria | 6 | Specify study characteristics (e.g., PICOS, length of follow-up) and report characteristics (e.g., years considered, language, publication status) used as criteria for eligibility, giving rationale. | 4 |
| Information sources | 7 | Describe all information sources (e.g., databases with dates of coverage, contact with study authors to identify additional studies) in the search and date last searched. | 4 |
| Search | 8 | Present full electronic search strategy for at least one database, including any limits used, such that it could be repeated. | Appendix S1 |
| Study selection | 9 | State the process for selecting studies (i.e., screening, eligibility, included in systematic review, and, if applicable, included in the meta-analysis). | 4-5 |
| Data collection process | 10 | Describe method of data extraction from reports (e.g., piloted forms, independently, in duplicate) and any processes for obtaining and confirming data from investigators. | 4-5 |
| Data items | 11 | List and define all variables for which data were sought (e.g., PICOS, funding sources) and any assumptions and simplifications made. | 4-5 |
| Risk of bias in individual studies | 12 | Describe methods used for assessing risk of bias of individual studies (including specification of whether this was done at the study or outcome level), and how this information is to be used in any data synthesis. | 5 & Appendix S2 |
| Summary measures | 13 | State the principal summary measures (e.g., risk ratio, difference in means). | 6 |
| Synthesis of results | 14 | Describe the methods of handling data and combining results of studies, if done, including measures of consistency (e.g., I^2^) for each meta-analysis. | 6-7 |

| **Section/topic** | **#** | **Checklist item** | **Reported on page #** |
| --- | --- | --- | --- |
| Risk of bias across studies | 15 | Specify any assessment of risk of bias that may affect the cumulative evidence (e.g., publication bias, selective reporting within studies). | NA |
| Additional analyses | 16 | Describe methods of additional analyses (e.g., sensitivity or subgroup analyses, meta-regression), if done, indicating which were pre-specified. | 6-7 |
| **RESULTS** | | |  |
| Study selection | 17 | Give numbers of studies screened, assessed for eligibility, and included in the review, with reasons for exclusions at each stage, ideally with a flow diagram. | Figure 1 |
| Study characteristics | 18 | For each study, present characteristics for which data were extracted (e.g., study size, PICOS, follow-up period) and provide the citations. | Table S2 in appendix |
| Risk of bias within studies | 19 | Present data on risk of bias of each study and, if available, any outcome level assessment (see item 12). | Table S2 in appendix |
| Results of individual studies | 20 | For all outcomes considered (benefits or harms), present, for each study: (a) simple summary data for each intervention group (b) effect estimates and confidence intervals, ideally with a forest plot. | Table 1 |
| Synthesis of results | 21 | Present results of each meta-analysis done, including confidence intervals and measures of consistency. | NA |
| Risk of bias across studies | 22 | Present results of any assessment of risk of bias across studies (see Item 15). | NA |
| Additional analysis | 23 | Give results of additional analyses, if done (e.g., sensitivity or subgroup analyses, meta-regression [see Item 16]). | 7-8 |
| **DISCUSSION** | | |  |
| Summary of evidence | 24 | Summarize the main findings including the strength of evidence for each main outcome; consider their relevance to key groups (e.g., healthcare providers, users, and policy makers). | 9-10 |
| Limitations | 25 | Discuss limitations at study and outcome level (e.g., risk of bias), and at review-level (e.g., incomplete retrieval of identified research, reporting bias). | 10-11 |
| Conclusions | 26 | Provide a general interpretation of the results in the context of other evidence, and implications for future research. | 11 |
| **FUNDING** | | |  |
| Funding | 27 | Describe sources of funding for the systematic review and other support (e.g., supply of data); role of funders for the systematic review. | 1 |

# References

1. Casalegno JS, Ottmann M, Bouscambert-Duchamp M, Valette M, Morfin F, Lina B. Impact of the 2009 influenza A(H1N1) pandemic wave on the pattern of hibernal respiratory virus epidemics, France, 2009. *Euro Surveill.* 2010;15(6):11.

2. Grondahl B, Ankermann T, von Bismarck P, et al. The 2009 pandemic influenza A(H1N1) coincides with changes in the epidemiology of other viral pathogens causing acute respiratory tract infections in children. *Infection.* 2014;42(2):303-308.

3. Hirsh S, Hindiyeh M, Kolet L, et al. Epidemiological Changes of Respiratory Syncytial Virus (RSV) Infections in Israel. *PLoS ONE.* 2014;9(3):e90515.

4. Mak GC, Wong AH, Ho WY, Lim W. The impact of pandemic influenza A (H1N1) 2009 on the circulation of respiratory viruses 2009-2011. *Influenza other respi.* 2012;6(3):e6-10.

5. Meningher T, Hindiyeh M, Regev L, Sherbany H, Mendelson E, Mandelboim M. Relationships between A(H1N1)pdm09 influenza infection and infections with other respiratory viruses. *Influenza other respi.* 2014;8(4):422-430.

6. Navarro-Mari JM, Perez-Ruiz M, Galan Montemayor JC, et al. Circulation of other respiratory viruses and viral co-infection during the 2009 pandemic influenza. *Enferm Infecc Microbiol Clin.* 2012;30 Suppl 4:25-31.

7. Yang L, Chan KH, Suen LK, et al. Impact of the 2009 H1N1 Pandemic on Age-Specific Epidemic Curves of Other Respiratory Viruses: A Comparison of Pre-Pandemic, Pandemic and Post-Pandemic Periods in a Subtropical City. *PLoS ONE.* 2015;10(4):e0125447.

8. Yang Y, Wang Z, Ren L, et al. Influenza A/H1N1 2009 pandemic and respiratory virus infections, Beijing, 2009-2010. *PLoS ONE.* 2012;7(9).

9. Ali A, Yousafzai MT, Waris R, et al. RSV associated hospitalizations in children in Karachi, Pakistan: Implications for vaccine prevention strategies. *J Med Virol.* 2017;89(7):1151-1157.

10. Althouse BM, Flasche S, Minh LN, et al. Seasonality of respiratory viruses causing hospitalizations for acute respiratory infections in children in Nha Trang, Vietnam. *Int J Infect Dis.* 2018;75:18-25.

11. Balmaks R, Ribakova I, Gardovska D, Kazaks A. Molecular epidemiology of human respiratory syncytial virus over three consecutive seasons in Latvia. *J Med Virol.* 2014;86(11):1971-1982.

12. Bruden DJ, Singleton R, Hawk CS, et al. Eighteen Years of Respiratory Syncytial Virus Surveillance: Changes in Seasonality and Hospitalization Rates in Southwestern Alaska Native Children. *Pediatr Infect Dis J.* 2015;34(9):945-950.

13. Mullins JA, Lamonte AC, Bresee JS, Anderson LJ. Substantial variability in community respiratory syncytial virus season timing. *Pediatr Infect Dis J.* 2003;22(10):857-862.

14. Buchan SA, Chung H, Karnauchow T, et al. Characteristics and outcomes of young children hospitalized with laboratory-confirmed influenza or respiratory syncytial virus in Ontario, Canada, 2009-2014. *Pediatr Infect Dis J.* 2019;38(4):362-369.

15. Callahan ZY, Smith TK, Ingersoll C, Gardner R, Korgenski EK, Sloan CD. Comparative Seasonal Respiratory Virus Epidemic Timing in Utah. *Viruses.* 2020;12(3):29.

16. Cui D, Feng L, Chen Y, et al. Clinical and Epidemiologic Characteristics of Hospitalized Patients with Laboratory-Confirmed Respiratory Syncytial Virus Infection in Eastern China between 2009 and 2013: A Retrospective Study. *PLoS ONE.* 2016;11(11):e0165437.

17. Cui G, Zhu R, Qian Y, et al. Genetic variation in attachment glycoprotein genes of human respiratory syncytial virus subgroups a and B in children in recent five consecutive years. *PLoS ONE.* 2013;8(9):e75020.

18. Dong L, Dai L, Fan J, et al. [Epidemiologic characteristics and the relationship with disease severity of respiratory syncytial virus genotypes from children with lower respiratory tract infection in the southern Zhejiang province]. *Zhonghua Er Ke Za Zhi.* 2015;53(7):537-541.

19. Feng L, Li Z, Zhao S, et al. Viral etiologies of hospitalized acute lower respiratory infection patients in China, 2009-2013. *PLoS ONE.* 2014;9(6).

20. Ferone EA, Berezin EN, Durigon GS, et al. Clinical and epidemiological aspects related to the detection of adenovirus or respiratory syncytial virus in infants hospitalized for acute lower respiratory tract infection. *J Pediatr (Rio J).* 2014;90(1):42-49.

21. Ferrero F, Torres F, Abrutzky R, et al. Seasonality of respiratory syncytial virus in Buenos Aires. Relationship with global climate change. *Arch Argent Pediatr.* 2016;114(1):52-55.

22. Donaldson GC. Climate change and the end of the respiratory syncytial virus season. *Clin Infect Dis.* 2006;42(5):677-679.

23. Gamba-Sanchez N, Rodriguez-Martinez CE, Sossa-Briceno MP. Epidemic activity of respiratory syncytial virus is related to temperature and rainfall in equatorial tropical countries. *Epidemiology and Infection.* 2016;144(10):2057-2063.

24. Gentile A, Lucion MF, Juarez MDV, et al. Burden of Respiratory Syncytial Virus Disease and Mortality Risk Factors in Argentina: 18 Years of Active Surveillance in a Children's Hospital. *Pediatr Infect Dis J.* 2019;38(6):589-594.

25. Hamada H, Ogura A, Hotta C, Wakui T, Ogawa T, Terai M. [Epidemiological study of respiratory viruses detected in patients under two years old who required admission because of lower respiratory disease]. *Kansenshogaku Zasshi.* 2014;88(4):423-429.

26. He Y, Lin GY, Wang Q, et al. A 3-year prospective study of the epidemiology of acute respiratory viral infections in hospitalized children in Shenzhen, China. *Influenza other respi.* 2014;8(4):443-451.

27. Horton KC, Dueger EL, Kandeel A, et al. Viral etiology, seasonality and severity of hospitalized patients with severe acute respiratory infections in the Eastern Mediterranean Region, 2007-2014. *PLoS ONE.* 2017;12(7):e0180954.

28. Kang SY, Hong CR, Kang HM, et al. Clinical and epidemiological characteristics of human metapneumovirus infections, in comparison with respiratory syncytial virus A and B. *Pediatric Infection and Vaccine.* 2013;20(3):168-177.

29. Kanou K, Arima Y, Kinoshita H, et al. Respiratory Syncytial Virus Surveillance System in Japan: Assessment of Recent Trends, 2008-2015. *Jpn J Infect Dis.* 2018;71(3):250-255.

30. Kim J, Jeon J, Kim J. Weather and its effects on RSV A and B infections in infants and children in Korea. *Australasian Medical Journal.* 2017;10(12):997-1002.

31. Liu J, Mu Y, Dong W, et al. Genetic variation of human respiratory syncytial virus among children with fever and respiratory symptoms in Shanghai, China, from 2009 to 2012. *Infect Genet Evol.* 2014;27:131-136.

32. Midgley CM, Haynes AK, Baumgardner JL, et al. Determining the Seasonality of Respiratory Syncytial Virus in the United States: The Impact of Increased Molecular Testing. *J Infect Dis.* 2017;216(3):345-355.

33. Naorat S, Chittaganpitch M, Thamthitiwat S, et al. Hospitalizations for acute lower respiratory tract infection due to respiratory syncytial virus in Thailand, 2008-2011. *J Infect Dis.* 2013;208 Suppl 3:S238-245.

34. Nyoka R, Achia TNO, Omony J, Musili SM, Gichangi A, Mwambi H. Time series non-Gaussian Bayesian bivariate model applied to data on HMPV and RSV: a case of Dadaab in Kenya. *BMC Public Health.* 2019;19(1):807.

35. Onozuka D. The influence of diurnal temperature range on the incidence of respiratory syncytial virus in Japan. *Epidemiol Infect.* 2015;143(4):813-820.

36. Reeves RM, Hardelid P, Gilbert R, Warburton F, Ellis J, Pebody RG. Estimating the burden of respiratory syncytial virus (RSV) on respiratory hospital admissions in children less than five years of age in England, 2007-2012. *Influenza other respi.* 2017;11(2):122-129.

37. Renko M, Tapiainen T. Change in respiratory syncytial virus seasonality in Finland. *Acta Paediatr.* 2020;109(1):202-203.

38. Ucakar V, Socan M, Trilar KP. The impact of influenza and respiratory syncytial virus on hospitalizations for lower respiratory tract infections in young children: Slovenia, 2006-2011. *Influenza other respi.* 2013;7(6):1093-1102.

39. Vos LM, Teirlinck AC, Lozano JE, et al. Use of the moving epidemic method (MEM) to assess national surveillance data for respiratory syncytial virus (RSV) in the Netherlands, 2005 to 2017. *Euro Surveill.* 2019;24(20).

40. Vega T, Lozano JE, Meerhoff T, et al. Influenza surveillance in Europe: establishing epidemic thresholds by the moving epidemic method. *Influenza Other Respir Viruses.* 2013;7(4):546-558.

41. Yu J, Liu C, Xiao Y, et al. Respiratory Syncytial Virus Seasonality, Beijing, China, 2007-2015. *Emerg Infect Dis.* 2019;25(6):1127-1135.

42. Canada Go. FluWatch. <https://open.canada.ca/en/open-data>. Published 2020. Accessed 10-Mar, 2021.

43. Japan National Institute of infectious diseases. Isolation & Detection of Viruses in the past years. <https://www.niid.go.jp/niid/en/typhi-m/iasr-reference/230-iasr-data/5495-iasr-table-ve-p.html>. Published 2015. Accessed 10-Mar, 2021.

44. New Zealand Ministry of Health. Virology Annual Report. <https://surv.esr.cri.nz/virology/virology_annual_report.php>. Published 2021. Accessed 10-Mar, 2021.

45. Li Y, Reeves RM, Wang X, et al. Global patterns in monthly activity of influenza virus, respiratory syncytial virus, parainfluenza virus, and metapneumovirus: a systematic analysis. *Lancet Glob Health.* 2019;7(8):e1031-e1045.

46. Li Y. *Global seasonality of respiratory viruses and the association between viral acute respiratory infection and subsequent pneumococcal disease*, University of Edinburgh; 2019.
